# An osseous lesion in a 10-year-old boy with Hodgkin's lymphoma: a case report

**DOI:** 10.1186/1752-1947-5-511

**Published:** 2011-10-08

**Authors:** Machiel van den Akker, Vadiem Zudekov, Asher Moser, Joseph Kapelushnik

**Affiliations:** 1 Department of Paediatric Haematology Oncology, Queen Paola Children's Hospital, 2020, Antwerpen, Belgium; 2 Radiology Department, University Medical Center Soroka, Beer Sheva, Israel; 3 Department of Paediatric Haematology Oncology, University Medical Center Soroka, Beer Sheva, Israel

## Abstract

**Introduction:**

Osseous involvement of Hodgkin's lymphoma is uncommon. When osteolytic lesions are seen on imaging it is important to evaluate potential other causes.

**Case presentation:**

We report the case of a 10-year-old Caucasian boy who presented to our facility with a bony lesion of the right clavicle and enlarged cervical lymph nodes. A simultaneous biopsy of the lymph node and of the osteolytic process of his right proximal clavicle was performed and revealed two different kinds of lesions: a mixed cellularity Hodgkin's lymphoma and an osteochondroma.

**Conclusions:**

Since the latter is a common benign bone tumor, which should not interfere with the staging of the lymphoma, we emphasize the importance of ensuring that all efforts are made to acquire a diagnostic biopsy of all atypical lesions.

## Introduction

Lymphoma is the third most common childhood malignancy following leukemia and brain tumors, accounting for approximately 12% of childhood cancers. Two-thirds of lymphomas diagnosed in children are non-Hodgkin's lymphomas (NHL), with the remainder being Hodgkin's lymphomas (HL). Anatomic extent of disease and tumor burden at presentation are significant factors determining choice of therapy and prognosis. HL typically involves the lymphatic system, and is usually supra-diaphragmatic. HL often follows a pattern of contiguous spread from one nodal group to the next anatomical region. Extra-nodal involvement is more common in NHL. Extra-nodal invasion of adjacent tissues is seen in up to 15% of cases and hematogenous spread in up to 10% of newly diagnosed cases. Osseous localizations have been described in 10% to 20% of cases of relapsed or refractory HL, but less than 2% at the time of initial presentation. Here, we describe a case of an osteochondroma (OC) in a child with Hodgkin's disease not affecting therapy or prognosis.

## Case presentation

Due to enlarged lymph nodes in his right neck region, a 10-year-old Caucasian boy underwent ultrasonic investigation and was treated with a short course of antibiotics 18 months prior to his presentation at our facility. Two months before his current admission, our patient reported local pain and enlargement of the same area in the neck. No B symptoms were evident. A second antibiotic treatment was prescribed, presuming a diagnosis of lymphadenitis at that time, and all laboratory tests were within the normal range except a slight microcytic hypochromic anemia (hemoglobin 10.8 g/dL, mean corpuscular volume 72).

**Figure 1:**
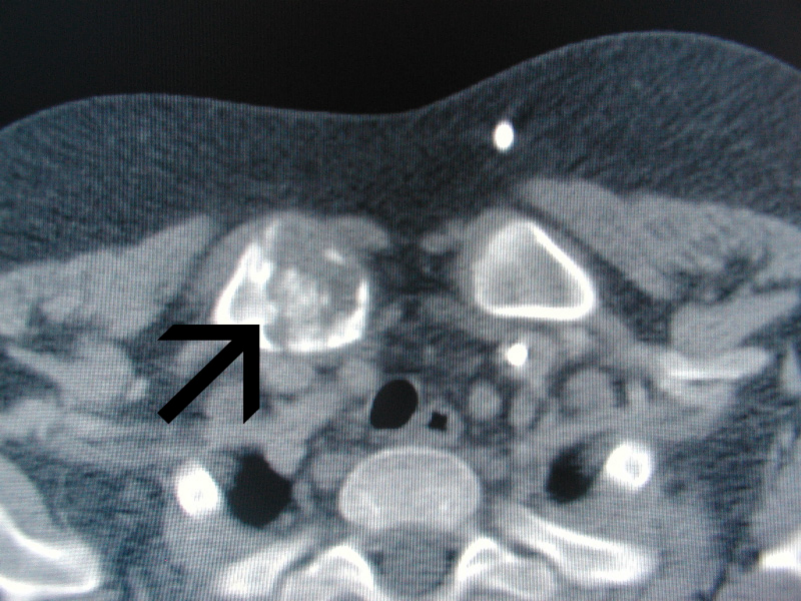
**Computed tomography scan showing an osteolytic process with periosteal reaction of the right proximal clavicle**.

Plain X-rays of the chest showed no abnormal findings. An ultrasonographic study of the neck showed enlarged lymph nodes (measuring up to 3.2 cm in diameter) on the right side, mostly in the posterior triangle. A computed tomography (CT) scan of the neck, thorax and abdomen confirmed a heterogeneous mass of enlarged lymph nodes on the right side of the neck and an osteolytic process accompanied by a periosteal (and soft-tissue) reaction in the right proximal clavicle, conspicuous for a tumor or chronic osteomyelitis (Figure 1). Radionuclide imaging with gallium-67 citrate showed pathologic absorption on the right side of the neck, in accordance with the enlarged lymph nodes, but not in the right proximal clavicle. A comparable study with fluorodeoxyglucose positron emission tomography (FDG-PET) was not available.

**Figure 2:**
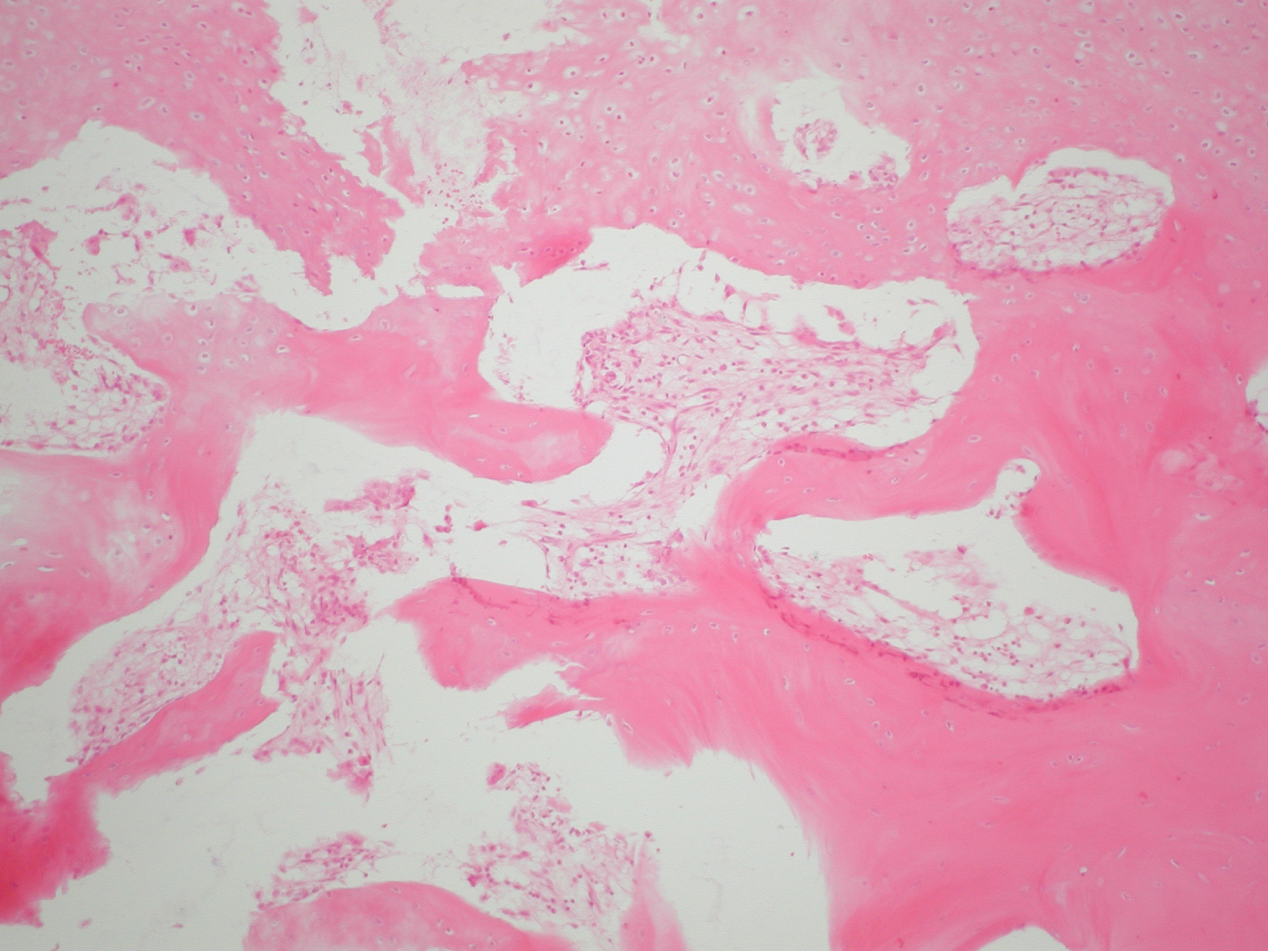
**Irregular fragment of a bone lesion showing cartilaginous cup and exophytic bone (hematoxylin and eosin staining, magnification ×250)**.

Biopsies of a lymph node in the posterior triangle on the right side of the neck and from the right proximal clavicle were taken. The biopsy of the lymph node confirmed the diagnosis of Hodgkin's lymphoma with mixed cellularity and a focal inter-follicular pattern. Immunohistochemistry stains were positive for CD15 and CD30. The clavicle biopsy showed bone tissue with exophytic cartilaginous tissue (Figure 2), consistent with osteochondroma without evidence of involvement with HL. Our patient underwent four courses of ABVD chemotherapy protocol (doxorubicin, bleomycin, vinblastine and dacarbazine) for stage IIa disease and is currently five years on from cessation of all treatment and in complete remission.

## Discussion

Osteocartilaginous exostoses (osteochondromas (OC)) are the commonest of all bone tumors, compromising about one-third of benign bone tumors. They may be sporadic, genetic or secondary (for example, from radiation). The majority are solitary, mostly located around the knee and in the proximal humerus, while about 20% arise in the axial skeleton. Typically, they are metaphyseal or metadiaphyseal in origin, oriented away from the adjacent joint. OC may occur at multiple sites, particularly in cases of hereditary multiple exostoses syndrome (HMES) with an autosomal dominant inheritance. Most children are asymptomatic. OC usually presents as a solitary mass, as a fracture or as a mechanical osteoarticular complication (deformity or joint dysfunction). Plain radiography is the mainstay of imaging for OC, while a CT scan can be helpful when planning resection. An MRI scan is needed when there are concerns of a malignancy. Bone scans are highly sensitive, but with low specificity, and are in general not useful. Histology is necessary to confirm a diagnosis of OC, showing a cartilage-capped exophytic, sessile or pedunculated projection that has a continuous peri-osteum, cortex and marrow connecting with the underlying bone. The cartilage cap is the site of active growth and the degree of maturity is parallel with the host bone. It is not common for OC to grow beyond skeletal maturity. The risk of malignant transformation to chondrosarcoma of a solitary OC is 1% to 5%, but for HMES the reported incidence is up to 9% [1]. Frequent sites are the pelvis, proximal femur and the shoulder girdle. The presence of an enlarging mass (especially after skeletal maturity) and recent onset of pain should arouse suspicion of malignant transformation. Treatment of OC is usually accomplished by resection or curettage, and rarely requires bone grafting.

Although presenting symptoms due to osseous involvement rarely occur in HL, osseous involvement is seen at radiography in approximately 20% of affected patients throughout the course of the disease [2]. Osseous involvement may be indicative of widespread, aggressive disease with a relative poor prognosis, associated with unfavorable histological subtypes. Primary osseous HL is very rare and must be distinguished from systemic HL with diffuse bone and bone marrow involvement and from osseous metastases in advanced stage of disease. Due to the nature of HL, most cases are associated with synchronous lymph node involvement. Bone scintigraphy has a sensitivity and accuracy of 95% in detecting osseous involvement, but in most cases bone HL is revealed on plain X-rays and CT scanning [3]. The roentgen graphic features of bony involvement of HL are non-specific, and may be solitary (33%) or polyostotic (66%) [4]; the edge is usually wide and ill defined, there may be a periosteal reaction with bone destruction, and the lesions are predominantly osteolytic with blurred borders [2]. Fractures are rarely the first manifestations. Soft-tissue tumors are often seen adjacent to the bone lesions. Differential diagnosis included primary sarcoma of the bone, NHL, leukemia, metastasis, and the most frequent (histopathologic and radiographic) misdiagnosis, osteomyelitis. In general, routine bone scintigraphy seems to be of limited value in the clinical assessment of children with malignant lymphoma unless there are specific osseous symptoms. FDG-PET has replaced the 67 Ga scintigraphy for evaluating children and younger adults with newly diagnosed HD [5]. Experience of OC is limited and is also not uniformly informative with regards to diagnostic investigation for malignant transformation.

## Conclusions

We describe a case of coinciding osseous lesion and HL. We conclude that all efforts should be taken to make an exact diagnosis by biopsy of all suspicious locations, including bony structures, in order to make an accurate diagnosis and subsequently start the appropriate treatment.

## Consent

Written informed consent was obtained from the patient's legal guardian for publication of this case report and any accompanying images. A copy of the written consent is available for review by the Editor-in-Chief of this journal.

## Competing interests

The authors declare that they have no competing interests.

## Authors' contributions

MA was responsible for the data collection, obtaining consent, and was the author of the manuscript. VD was responsible for the imaging and part of the Discussion section. AM was responsible for carefully reviewing the article. JK was responsible for the medical care of our patient and for part of the Discussion section. All authors read and approved the final manuscript.
